# From static prediction to dynamic cancer trajectories: Virtual Human Twins for breast cancer decision support

**DOI:** 10.1371/journal.pdig.0001548

**Published:** 2026-07-09

**Authors:** Yen Y. Tan, Ivana Janickova, Georg Langs

**Affiliations:** 1 Department of Obstetrics and Gynecology, Comprehensive Cancer Center, Medical University of Vienna, Vienna, Austria; 2 Computational Imaging Research Lab, Department of Biomedical Imaging and Image-guided Therapy, Medical University of Vienna, Vienna, Austria; 3 Comprehensive Center for Artificial Intelligence in Medicine, Medical University of Vienna, Vienna, Austria; 4 Christian Doppler Laboratory for Machine Learning Driven Precision Imaging, Department of Biomedical Imaging and Image-guided Therapy, Medical University of Vienna, Vienna, Austria; Ben-Gurion University of the Negev, ISRAEL

## Abstract

Digital oncology has become increasingly sophisticated at predicting cancer risk, treatment response, and prognosis. Yet many tools still operate as static snapshots, while cancer care unfolds as a trajectory shaped by tumor biology, genetics, treatment, residual disease, toxicity, and patient priorities. We argue that oncology needs dynamic, multiscale Virtual Human Twins (VHTs) that represent the patient and disease as they evolve together, integrating multimodal data to support longitudinal clinical reasoning and decision simulation rather than prediction alone. High-risk triple-negative breast cancer provides a focused first use case because trajectories are heterogeneous, decisions are time-sensitive, and clinical utility can be tested in tumor-board workflows. A minimum viable VHT should make uncertainty visible, support clinically interpretable simulation of alternative management strategies, and be judged by clinical decision impact, not predictive performance alone.

## Introduction

Digital oncology has become increasingly sophisticated at predicting risk, treatment response, and prognosis [1,2]. Yet prediction alone has not changed the operating model of cancer care. Many tools still generate estimates at a defined clinical moment: a genetic test result, baseline tumor assessment, radiological response, pathology after surgery, recurrence probability, or survivorship plan [3]. These snapshots are clinically important, but they do not reflect how cancer behaves, or how patients live through cancer. Cancer unfolds as a trajectory shaped by inherited susceptibility, tumor biology, treatment, residual disease, recurrence risk, toxicity, survivorship needs, and changing patient priorities [4–8].

The next step for digital health in oncology is therefore not simply to build more accurate models, but to develop Virtual Human Twins (VHTs) that can represent the patient and disease as they evolve together [2,9]. Their value should not lie in producing a more elaborate risk calculator, but in helping clinicians understand why trajectories diverge, simulate how they may change under alternative strategies, and make possible futures visible enough to support clinical decision-making [1,2,9].

This distinction matters because a VHT is not simply a digital twin, a computational patient model, a longitudinal risk predictor, or a foundation-model copilot for clinical summarization [2,9,10]. These are complementary components or enabling technologies that may contribute to a VHT, but they do not by themselves constitute one. A digital twin may provide a synchronized representation of selected biological or clinical processes [11], a computational patient model may simulate specific physiological mechanisms, a longitudinal risk predictor estimates future outcomes from evolving data, and a foundation-model copilot assists with information synthesis or clinical reasoning. By contrast, a VHT is a clinically bounded, patient-specific, longitudinal whole-person model. It continuously integrates clinical, imaging, molecular, genetic, treatment, lifestyle, and patient-reported data to maintain an evolving representation of the patient and disease over time [1,2]. It captures the dynamic interaction between tumor biology, inherited susceptibility, treatment exposure, patient health state, uncertainty and patient-centered outcomes, and uses this representation to explore clinically plausible future trajectories and management options within a defined clinical workflow [2,9,12]. Its distinguishing feature is not the aggregation of more data or the use of more sophisticated prediction, but the creation of a continuously updated, interpretable computational representation that supports clinician-in-the-loop reasoning, counterfactual simulation and decision-making across the cancer journey [9,12,13].

## VHTs as decision-simulation systems

We propose multimodal clinical reasoning and decision simulation as the translational layer required for oncology VHTs. By this, we mean a longitudinal digital representation of a patient’s evolving health and disease state, maintained over time within an in-silico environment that integrates multimodal data and compares clinically plausible, guideline-concordant options under explicit uncertainty. This is distinct from a static prediction model or conventional digital twin framework. It is not autonomous treatment selection, but a clinician-in-the-loop environment for asking what has changed in this patient’s trajectory, which options remain reasonable, how these options may alter the projected trajectory, what evidence supports them, what trade-offs are expected, and how these align with the patient’s priorities.

**Fig 1:**
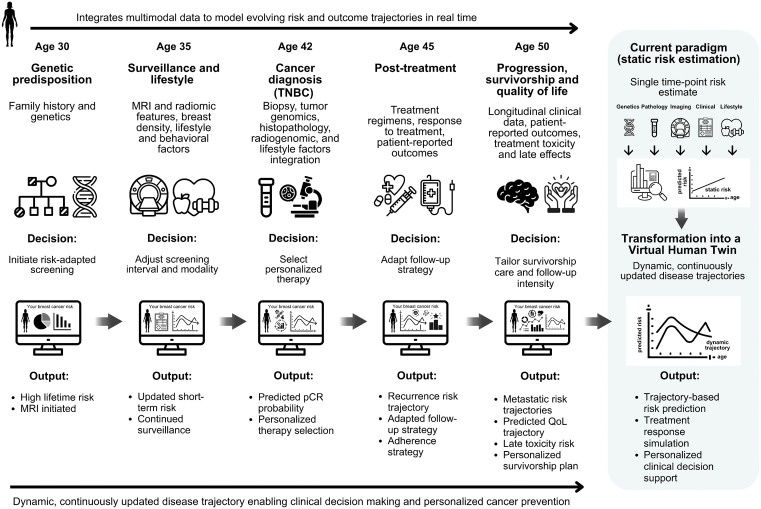
Dynamic, multiscale Virtual Human Twin framework for breast cancer decision support. The figure is created by the authors using Canva.

Breast cancer is an appropriate setting for this shift because it is data-rich, but not yet continuously modeled. Imaging, pathology, genomics, treatment history, symptoms, guidelines, and patient circumstances are often interpreted in separate clinical moments rather than as part of a continuous disease trajectory. Multidisciplinary tumor boards bring these data together under real constraints: time pressure, incomplete information, evolving evidence, and the difficulty of weighing survival benefit, toxicity, quality of life, and patient preference simultaneously [14,15]. The challenge is not lack of information, but fragmentation across the patient trajectory. Fig 1 illustrates the proposed longitudinal VHT framework.

This ambition also exposes a central limitation of VHT development. The challenge is not only algorithmic performance, but the construction of analysis-ready longitudinal data across systems that were not designed to interoperate. Radiology, pathology, genomics, electronic health records, treatment data, and patient-reported outcomes differ in format, timing, granularity, completeness, consent status and clinical meaning. A credible VHT will therefore require explicit data models, common vocabularies, temporal alignment, provenance tracking, quality control, strategies for missing or inconsistent data, calibration monitoring, subgroup validation and governance for model updating [9,11,13,16–18]. Without these foundations, a VHT risks becoming an elegant interface layered over fragmented, biased, or clinically incomplete data.

## Why breast cancer, and why high-risk TNBC?

High-risk triple-negative breast cancer (TNBC) illustrates this challenge clearly. TNBC is aggressive, heterogeneous, and clinically time-sensitive. Patients with apparently similar tumor subtype, stage, and treatment exposure may experience markedly different paths: pathological complete response and durable remission in one patient; residual disease, early recurrence, organ-specific metastasis, or long-term treatment-related morbidity in another [19,20]. *BRCA1/2*-associated and homologous recombination deficiency-driven biology provides a mechanistic foundation for part of this divergence, linking inherited susceptibility, DNA repair deficiency, treatment sensitivity, residual disease, and metastatic potential. Risk models such as BOADICEA/CanRisk have substantially advanced individualized risk estimation in *BRCA1* and *BRCA2* pathogenic variant carriers [5,21]. Yet once a young *BRCA1* carrier develops node-positive TNBC, lifetime risk becomes secondary to the connected decisions made under uncertainty. Which neoadjuvant strategy should be considered? Is response sufficient, or should treatment be adapted? Does residual disease imply escalation? When might de-escalation be safe? How should surgery, fertility preservation, menopause management, surveillance, behavioral factors, and survivorship care be integrated into a coherent long-term plan? Yet biology alone does not determine outcome. Clinical trajectories are shaped by treatment exposure, response dynamics, comorbidity, symptom burden, adherence, lifestyle and behavioral factors, and individual survivorship priorities.

## What a minimum viable VHT should do

A minimum viable VHT should be focused, clinically grounded, and testable. It does not need to model all of breast cancer from the outset; high-risk TNBC offers a well-defined setting to evaluate clinical utility and implementation. Foundational components are already emerging [1,22]. Yet these methods become clinically meaningful as a VHT only when integrated to support longitudinal clinical reasoning, transparent uncertainty, and decision simulation at meaningful points in the patient’s care trajectory.

Patient-reported outcomes are not optional in this framework. Randomized studies have shown that systematic electronic symptom monitoring can improve quality of life, reduce acute care use, and improve survival [23,24]. Symptoms, fatigue, pain, anxiety, fertility concerns, treatment burden, adherence, work participation, and functional recovery are part of the patient state, shaping what treatments can be tolerated, completed, and sustained.

Initial deployment should focus on these decision points. Outputs must be clinically legible. Clinicians and patients should be able to see how the estimated trajectory has changed, what evidence supports that change, how uncertainty is represented, and how alternative strategies compare. Updated trajectory estimates, patient-similarity views, evidence links, uncertainty ranges, and treatment trade-off dashboards are not merely interface features but prerequisites for trust and clinical adoption.

## From model performance to clinical utility

Evaluation must be held to the same standard. Predictive performance is necessary, but not enough. A VHT system should be judged by whether it improves interpretation and decision-making at clinically meaningful moments: calibration over time, recognition of divergent trajectories, subgroup validity, tumor-board usability, shared decision-making, timeliness of treatment adaptation, and patient-centered outcomes. Evaluation should include the infrastructure layer as well as the model layer, because a system that cannot be updated, audited, calibrated, or integrated into tumor-board workflow has not achieved clinical usefulness. Retrospective and external validation are essential first steps, but clinical utility will require tumor-board simulations and prospective clinician-in-the-loop studies [12]. The relevant question is not simply whether a model predicts better, but whether it helps clinicians and patients make better decisions.

The future of digital oncology should not be judged by increasingly sophisticated static prediction alone. VHTs should be evaluated by whether they improve care at the points where decisions are most consequential: when early response is uncertain, residual disease changes prognosis, escalation or de-escalation is being considered, symptoms threaten treatment completion, or survivorship priorities begin to compete with tumor-directed goals. Their clinical value lies in making changing trajectories visible before decisions are fixed, clarifying uncertainty, and supporting patient-centered trade-offs in real workflows. The goal is not to replace tumor boards or clinical judgment, but to help clinicians and patients reason from the whole trajectory: not only the tumor, but the person moving through treatment, recovery, recurrence risk, and survivorship. Every tumor follows a trajectory. Every patient lives through one. Digital systems should support both.
